# Functional Connectivity Changes in Primary Motor Cortex Subregions of Patients With Obstructive Sleep Apnea

**DOI:** 10.1002/brb3.70698

**Published:** 2025-07-25

**Authors:** Lifeng Li, Qimeng Shi, Bowen Fang, Yuting Liu, Xiang Liu, Yongqiang Shu, Yingke Deng, Yumeng Liu, Haijun Li, Junjie Zhou, Dechang Peng

**Affiliations:** ^1^ Jiangxi Provincial Key Laboratory for Precision Pathology and Intelligent Diagnosis, Department of Radiology, The First Affiliated Hospital, Jiangxi Medical College Nanchang University Nanchang China; ^2^ Department of Radiology, The Affiliated Changsha Central Hospital, Hengyang Medical School University of South China Changsha China; ^3^ School of Medical Imaging Hangzhou Medical College Hangzhou China; ^4^ Hengyang Medical School University of South China Hengyang China; ^5^ Department of Ophthalmology Hunan Children's Hospital Changsha China; ^6^ PET Center The First Affiliated Hospital of Nanchang University Nanchang China

**Keywords:** functional connectivity, obstructive sleep apnea, primary motor cortex, subregion, support vector machine

## Abstract

**Background and Purpose:**

Obstructive sleep apnea (OSA) is linked to cognitive impairment and altered motor‐related brain networks. This study examined functional connectivity (FC) changes in subregions of the primary motor cortex (M1) in patients with OSA and their association with sleep structure, cognition, and clinical features.

**Methods:**

Sixty‐five patients with OSA and 65 healthy controls (HC) participants matched in age and educational background were included. Resting‐state functional MRI data were acquired for all participants using a 3T MRI system. Based on the Human Brainnetome Atlas, we analyzed FC changes of 12 subregions of M1 across the whole brain in patients with OSA. The two‐sample *t*‐tests were conducted to compare FC values between subregions of M1 and other brain regions in two groups. Partial correlation analyses examined the association between FC and clinical variables in patients with OSA. Additionally, we employed three machine learning algorithms—support vector machine (SVM), random forest (RF), and logistic regression (LR)—to distinguish patients with OSA from HC based on FC features.

**Results:**

Compared to HC, the OSA group found that significant FC enhancements were identified in right A6cdl with the left inferior parietal lobule (IPL); left A4tl with the left inferior frontal gyrus (IFG), bilateral middle frontal gyrus (MFG), and left IPL; and left A6cvl with the right parahippocampal gyrus, bilateral MFG, left IFG, left superior temporal gyrus, and right cingulate gyrus. After Bonferroni correction, a negative correlation was observed between the FC value of A4tl (L)‐IPL (L) and N2 (*p* < 0.05). Furthermore, SVM yielded the highest area under the receiver operating characteristic (ROC) curve (AUC) among all classifiers, indicating its superior performance in discriminating OSA patients from HC based on FC features.

**Conclusion:**

The study demonstrates that OSA significantly impacts brain functional networks, particularly affecting motor control through altered FC in subregions of M1. These alterations correlate with upper airway dysfunction and cognitive impairments, increasing accident risks. The high‐accuracy SVM classification based on FC patterns demonstrates potential as a diagnostic biomarker for OSA. Future research should explore M1 FC patterns as diagnostic markers and neuromodulation therapies.

## Graphical abstract

This study revealed that OSA alters FC in primary motor cortex subregions, impairing motor control. These changes are linked to upper airway dysfunction and cognitive deficits. Among three machine learning models, support vector machine showed the best performance in classifying OSA, highlighting FC features as potential neuroimaging biomarkers.Special thanks to Figdraw (www.figdraw.com) for their support in creating the graphical abstract.

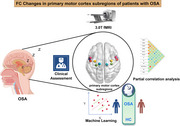

## 1 Introduction

Obstructive sleep apnea (OSA) is a common and serious sleep‐related breathing disorder characterized by repeated upper airway collapse during sleep, leading to episodes of apnea or hypoventilation. The global prevalence of OSA is increasing, currently affecting approximately 1 billion adults worldwide (Rokou et al. 2023). OSA not only severely compromises sleep quality but is also strongly associated with various health conditions, including cardiovascular diseases (e.g., hypertension and coronary heart disease), metabolic disorders (e.g., diabetes mellitus), and neurological issues such as cognitive dysfunction and mood disturbances (Gabryelska et al. 2024; Osman et al. 2018). Of particular concern is the potential impact of OSA on brain function, specifically its effects on brain functional connectivity (FC) (Li, Long, et al. 2024). Intermittent hypoxia, disrupted sleep architecture, and chronic fatigue in patients with OSA can significantly impair the integrity of functional brain networks, which not only affect cognitive function but may also interfere with motor control systems (Li, Long, et al. 2024). Moreover, studies have shown that patients with OSA are at a significantly elevated risk of motor vehicle accidents, experiencing rates that are two to seven times greater than those of the general population (Alqurashi et al. 2024; Minhas et al. 2024; Udholm et al. 2022). The impairment of the primary motor cortex and related motor control regions in patients with OSA may lead to delayed reaction times, impaired vigilance, and compromised motor coordination (Yaouhi et al. 2009). These motor control deficits have resulted in severe consequences, with OSA‐related traffic accidents accounting for approximately 7%–30% of all motor vehicle accidents and causing significant mortality, morbidity, and economic burden (Garbarino et al. 2015; Sassani et al. 2004). Therefore, investigating the impact of OSA on brain areas linked to motor control is essential to better understand the neuropathological mechanisms of this disease and its clinical manifestations.

The primary motor cortex (M1) is a core region of the motor control system that directs voluntary movements by transmitting motor commands to various body parts. It exerts direct control over skeletal muscles through the corticospinal tract, enabling precise and fine motor control (Kinoshita et al. 2024). However, M1 is not functionally homogeneous; it is composed of multiple specialized subregions, each controlling specific body parts such as the hand, face, or trunk. This refined functional organization allows M1 to modulate the movements of different body parts with a high degree of precision (He et al. 2023). Given M1's role in controlling various muscle groups and its functional organization (Massé‐Alarie et al. 2017), alterations in M1 and its FC in patients with OSA may contribute to their observed motor deficits, including prolonged reaction times, diminished fine motor skills, and reduced motor coordination, potentially explaining their increased risk of accidents (Garofalo et al. 2024). However, the neural mechanisms underlying motor‐related symptoms in OSA, particularly those involving M1 organization and its network interactions, remain unclear. Understanding the FC patterns of M1 may provide new insights into these mechanisms.

The FC refers to the statistical correlation of neural activity time series between different brain regions, reflecting the functional integration and information exchange that underpin cognitive and motor functions. In neuroimaging research, resting‐state functional magnetic resonance imaging (rs‐fMRI) has emerged as a primary tool for investigating FC abnormalities across various diseases because of its noninvasive nature and high temporal and spatial resolution (Geng et al. 2024). In the context of OSA, intermittent hypoxia and repeated awakenings disrupt cortical FC, particularly affecting motor‐related regions, which can directly impair motor control. Studies have shown that FC disturbances in OSA extend to cortical and subcortical regions, including the cerebellum, basal ganglia, and M1. As M1 governs muscle activation and movement through direct connections to spinal motor neurons, it also receives essential inputs from other motor‐related cortical (premotor cortex, supplementary motor area, and parietal cortex) and subcortical (basal ganglia and cerebellum) structures (Alcock et al. 2023). However, the specific impact of OSA on functional subregions of M1, and consequently on brain‐wide FC, remains underexplored. This gap is particularly notable in detailed rs‐fMRI analyses of the FC and associations within subregions of M1. Given that subregions of M1 play distinct roles in motor control due to their functional compartmentalization, a detailed examination of the FC between these subregions of M1 and the whole brain is crucial to uncover the specific effects of OSA on motor control and associated neural networks (Larivière et al. 2018). Beyond fMRI studies, other non‐PSG‐based approaches—particularly those using electroencephalography (EEG)—have also shown promise in distinguishing OSA from healthy individuals. Nassehi et al. (2024) extracted multiple‐domain features from awake EEG recordings and achieved high classification performance, while Tanci and Hekim (2025) used EEG spectrograms with deep learning models for sleep apnea detection. These EEG‐based methods underscore the growing interest in accessible, signal‐driven diagnostics. However, EEG is primarily limited to cortical surface activity and lacks the spatial resolution needed to assess deep or distributed brain networks. In contrast, our rs‐fMRI approach provides a complementary perspective by capturing spatially distributed FC alterations, particularly in motor‐related regions such as M1. Moreover, support vector machines (SVM), as a robust machine learning approach, can construct optimal classification boundaries in a high‐dimensional space, effectively distinguishing data by maximizing class separation (G. Chen et al. 2023; Li et al. 2023). The SVM algorithm is especially advantageous when working with high‐dimensional data and small sample sizes, and it has shown promise in the diagnostic modeling of neuropsychiatric disorders, including affective disorders, thought disorders, and neurodegenerative diseases. In addition, random forest (RF), a powerful ensemble learning method, enhances classification performance by aggregating multiple decision trees to reduce variance and improve generalization (Taye et al. 2025). Logistic regression (LR), known for its simplicity and interpretability, remains a reliable baseline model widely applied in clinical machine‐learning classification tasks (Zolnoori et al. 2023). Together with SVM, these models complement each other, enabling a more comprehensive investigation of FC patterns in OSA. By applying multiple machine learning models, including SVM, RF, and LR, this study aims to comprehensively explore FC patterns and identify distinct neural signatures associated with OSA (Berardi et al. 2023; Jiang et al. 2024; Tripathi et al. 2023).

**FIGURE 1:**
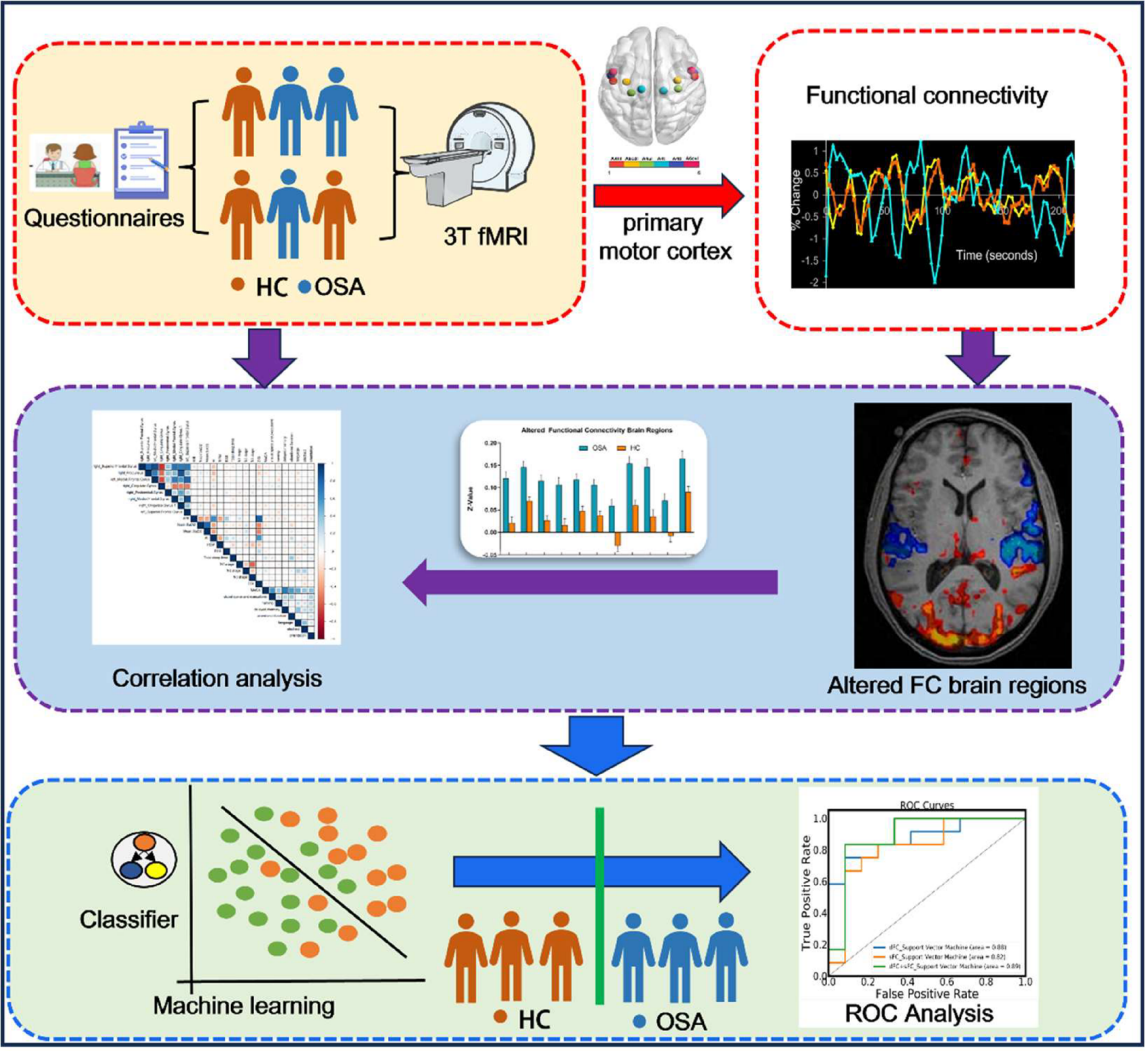
Flowchart illustrating the main steps of data processing and analysis for obstructive sleep apnea patients and healthy controls. HC, healthy controls; OSA, obstructive sleep apnea; ROC, receiver operating characteristic.

Based on this background, this study aimed to systematically explore resting‐state FC changes between subregions of M1 and other brain regions in patients with OSA using a combination of rs‐fMRI and SVM classification methods. We focused on how OSA may affect the FC of specific subregions of M1. We hypothesized that the FC in subregions of M1 would show significant alterations in patients with OSA and that these changes may be closely related to motor control impairments, disrupted sleep architecture, and cognitive dysfunction observed in these patients. To test this hypothesis, we employed rs‐fMRI along with seed‐point‐based FC analysis to examine the differences in FC in subregions of M1 between patients with OSA and healthy controls (HC). Additionally, we utilized SVM classification to distinguish patients with OSA from HC based on these FC differences. By systematically examining the unique impact of OSA on FC in subregions of M1, we aimed to deepen our understanding of the influence of OSA on the motor control system. As shown in Figure 1, the flowchart outlines the main steps of our methodology. Such insights may also provide novel functional imaging biomarkers that can be used in clinical diagnosis, prognostic assessment, and intervention strategies.

## 2 Materials and Methods

### 2.1 Participants

All participants were recruited from the Department of Respiratory or Otorhinolaryngology Sleep Monitoring Room of the First Affiliated Hospital of Nanchang University between July 2020 and July 2024. The final sample included 65 untreated patients with OSA and 65 HC, matched for age and educational background. Seventeen individuals (10 OSA and seven HC) were excluded based on the criteria described below. Inclusion criteria for the OSA group, based on the guidelines of the American Academy of Sleep Medicine (AASM) (Kapur et al. 2017), were as follows: (1) age between 18 and 60 years, (2) right‐handedness, and (3) an apnea–hypopnea index (AHI) ≥ 15 h^−1^. The HC group was defined as individuals with AHI < 5 h^−1^. Exclusion criteria for both groups included the following: (1) a history of hypertension, heart disease, or diabetes; (2) any central nervous system disorders (e.g., neurodegenerative diseases, psychosis, epilepsy, head trauma, or current depression); (3) brain MRI findings indicating structural abnormalities (e.g., cysts or tumors); (4) contraindications to MRI scanning (e.g., claustrophobia or metal implants); and (5) a history of drug or psychotropic substance abuse. This study was approved by the Ethics Committee of the First Affiliated Hospital of Nanchang University (approval No. 2020(12‐94)) and conducted in accordance with the Declaration of Helsinki. Written informed consent was obtained from all participants.

### 2.2 Overnight Polysomnography (PSG)

Each participant underwent a comprehensive overnight PSG monitoring, which included the recording of various physiological parameters. Before the sleep monitoring session, participants were instructed to abstain from hypnotics, alcohol, and caffeinated beverages. The PSG data were collected using the Respironics LE‐Series Physiological Monitoring System (Alice 5 LE; Respironics, Orlando, FL, USA). Also, electrocardiogram (ECG), standard EEG recording, chin electromyography (EMG), electrooculogram (EOG), thoracic and abdominal respiratory movements, and oxygen saturation (SaO_2_) were measured. PSG data were recorded from 10:00 p.m. to 6:00 a.m. the following morning. Following AASM guidelines (Kapur et al. 2017), OSA was defined as a ≥90% reduction in airflow sustained for at least 10 s; hypopnea was characterized as a ≥30% reduction in airflow lasting at least 10 s, accompanied by a SaO_2_ reduction of ≥3% and/or EEG arousal. The AHI was calculated as the average number of apnea and hypopnea events per hour of sleep, with OSA defined as an AHI of 5 or higher.

### 2.3 Clinical and Neuropsychological Measures

Each participant completed the Chinese versions of the Epworth Sleepiness Scale (ESS) and Pittsburgh Sleep Quality Index (PSQI) to evaluate daytime sleepiness and sleep quality. Cognitive function was assessed using the Montreal Cognitive Assessment (MoCA), while emotional function was evaluated using the Hamilton Anxiety Scale (HAMA) and Hamilton Depression Scale (HAMD). The ESS required participants to rate their likelihood of falling asleep in eight different scenarios using a progressively increasing probability scale ranging from 0 to 3. The scale yields a total score of 24, with scores above 6 indicating *drowsiness*, scores above 11 denoting *excessive drowsiness*, and scores exceeding 16 indicating *dangerous drowsiness*. The PSQI, which has a maximum score of 21, was used to measure sleep quality, with higher scores indicating poorer sleep quality. The MoCA comprises eight cognitive domains: executive functioning, attention and concentration, abstract thinking, memory, language, visuospatial abilities, calculations, and orientation. A maximum score of 30 is attainable, with scores below 26 indicating mild cognitive impairment. One point was added if the participant had less than 12 years of education to reduce educational bias (Nasreddine et al. 2005). The HAMD and HAMA scales assessed depressive and anxiety symptoms, respectively. A total HAMD score > 24 indicated *severe depression*, 17–24 indicated *mild to moderate depression*, and < 7 indicated *no depressive symptoms*. Similarly, a HAMA score > 29 indicated *severe anxiety*, 21–29 indicated *significant anxiety*, and < 7 indicated *no anxiety symptoms*. All assessments were conducted by professionally trained, blinded physicians, with tests administered in a consistent order to maintain reliability. All the scales above were utilized in this study with the appropriate copyright permissions obtained.

### 2.4 Rs‐fMRI Data Acquisition

Magnetic resonance data were obtained from all participants at the First Affiliated Hospital of Nanchang University using a 3.0T MRI scanner (Siemens, Erlangen, Germany) scanner with an eight‐channel phased‐array head coil. During scanning, foam was provided to reduce human‐induced head movements, and earmuffs to reduce noise interference from the scanner. All participants were instructed to relax, maintain stillness, keep their eyes closed as much as possible without falling asleep, and avoid active thought. Initially, routine T1‐ and T2‐weighted images were acquired to exclude any structural brain lesions that could potentially confound the results. Rs‐fMRI data were acquired with a gradient echo‐planar imaging (EPI) sequence. The imaging parameters were as follows: echo time (TE) = 30 ms, repetition time (TR) = 2000 ms, flip angle = 90°, slice thickness = 4.0 mm, interslice gap = 1.2 mm, field of view (FOV) = 230 × 230 mm^2^, matrix = 64 × 64, number of slices = 30, and total scan duration = 8 min. During acquisition, participants were instructed to lie still, keep their eyes closed, stay awake, and avoid active thinking. High‐resolution T1‐weighted structural images were acquired using a magnetization‐prepared rapid gradient echo sequence. Parameters were as follows: TE = 2.26 ms, TR = 1900 ms, flip angle = 9°, slice thickness = 1.0 mm, interslice gap = 0.5 mm, FOV = 250 × 250 mm^2^, matrix = 256 × 256, and number of slices = 176. Following the scans, two senior radiologists confirmed that none of the participants had any visible brain lesions.

### 2.5 Data Preprocessing

Data preprocessing was conducted using SPM12 (https://www.fil.ion.ucl.ac.uk/spm/software/spm12/) and DPABI (http://rfmri.org/dpabi) toolboxes on the MATLAB R2018b platform (MathWorks, USA). Initially, the quality of all MRI scans was assessed using MRIcro, and incomplete or corrupted data were excluded. For each participant, the first 10 volumes of functional images were discarded to allow for signal stabilization and minimize the effects of magnetic saturation. The remaining 230 volumes underwent slice timing correction and realignment to correct for head motion. Participants with head motion exceeding 2.0 mm in any direction or 2.0° in rotation were excluded. Each subject's T1‐weighted structural image was coregistered to the mean functional image, followed by tissue segmentation into gray matter, white matter, and cerebrospinal fluid using SPM12. Spatial normalization to the Montreal Neurological Institute space was then performed, and functional images were resampled to a voxel size of 3 × 3 × 3 mm^3^. Nuisance covariates—including 24 head motion parameters, global signal, and signals from white matter and cerebrospinal fluid—were regressed out. Finally, temporal band‐pass filtering (0.01–0.08 Hz) was applied to reduce low‐frequency drift and high‐frequency physiological noise.

### 2.6 FC Analysis

**FIGURE 2:**
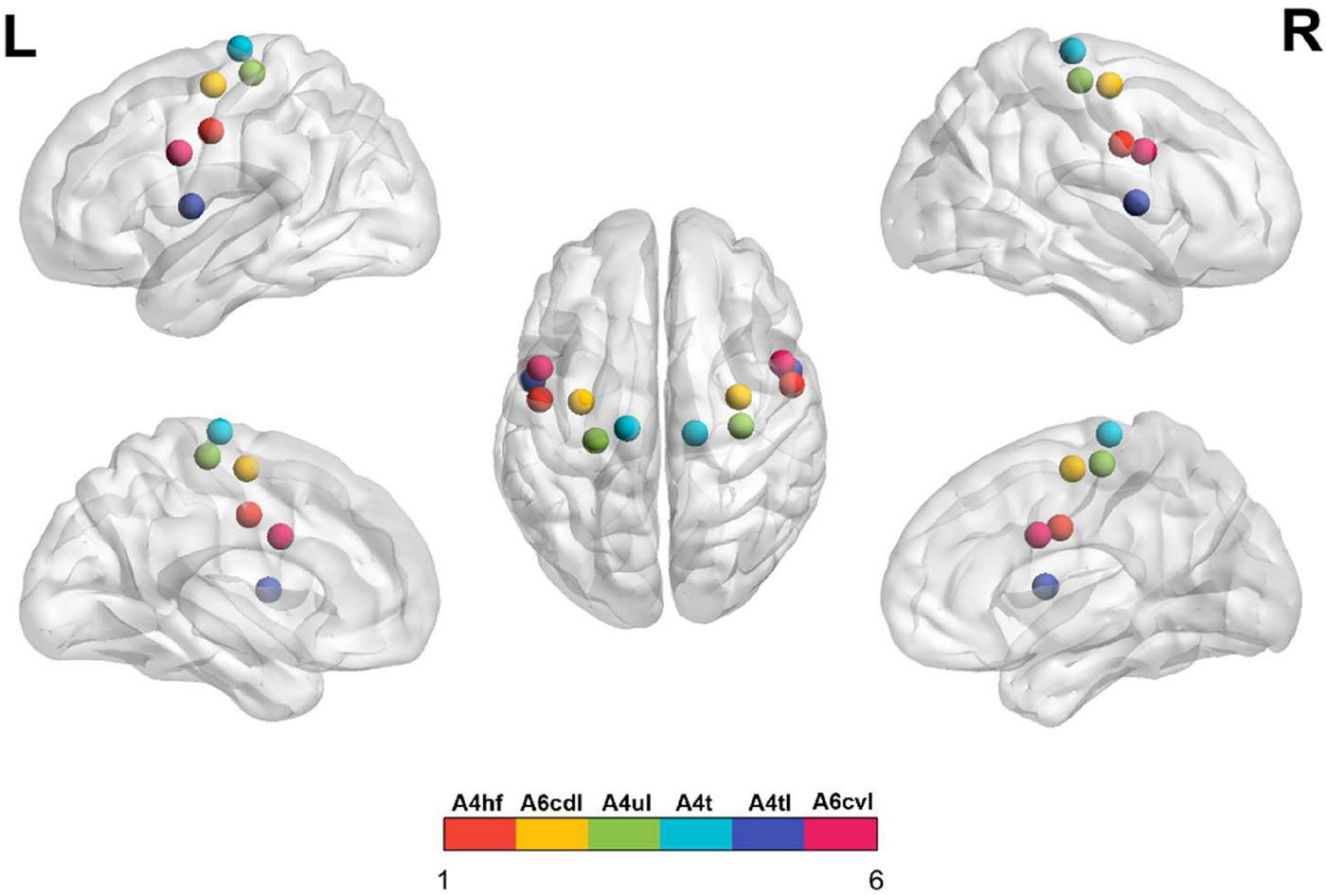
The 12 primary motor cortex subregions, including bilateral A4hf, A6cdl, A4ul, A4t, A4tl, and A6cvl.

The subregions of M1 were defined according to the Human Brainnetome Atlas which was based on the connectional architecture and applies multimodal neuroimaging techniques (Fan et al. 2016). In each hemisphere, the M1 was divided into six subregions: A4hf (head and face region in area 4), A6cdl (caudal dorsolateral area 6), A4ul (upper limb region in area 4), A4t (trunk region in area 4), A4tl (tongue and larynx region in area 4), and A6cvl (caudal ventrolateral area 6). Therefore, bilateral M1 included a total of 12 regions of interest (ROI). Detailed coordinates for these ROIs are shown in Figure 2 and Table 1.

**TABLE 1:** Detailed information of primary motor cortex subregions within the Human Brainnetome Atlas.

ROI (L/R)	Subregions	L_MNI (*X*, *Y*, *Z*)	R_MNI (*X*, *Y*, *Z*)
ROI 1,2	A4hf, area 4 (head and face region)	−49, −8, 39	55, −2, 33
ROI 3,4	A6cdl, area 6 (caudal dorsolateral)	32, −9, 58	33, −7, 57
ROI 5,6	A4ul, area 4 (upper limb region)	−26, −25, 63	34, −19, 59
ROI 7,8	A4t, area 4 (trunk region)	−13, −20, 73	15, −22, 71
ROI 9,10	A4tl, area 4 (tongue and larynx region)	−52, 0, 8	54, 4, 9
ROI 11,12	A6cvl, area 6 (caudal ventrolateral)	−49, 5, 30	51, 7, 30

Abbreviations: L, left; MNI, Montreal Neurological Institute; R, right; ROI, region of interest.

For the analysis, mean time series were first extracted from each ROI to serve as seeds for whole‐brain voxel‐wise FC analysis. Next, Pearson's correlation coefficients were computed between the ROI mean time series of each seed and all other gray matter voxels, resulting in whole‐brain seed‐based FC maps representing FC patterns in subregions of M1. These correlation maps were subsequently converted into *z*‐scores using Fisher's *r*‐to‐*z* transformation to improve data normality. Finally, the *z*‐transformed FC maps were smoothed with a 6 mm full‐width at half‐maximum Gaussian kernel. BrainNet Viewer was used to visualize the FC patterns across different brain regions.

### 2.7 Machine Learning Classification Analysis

The observed differences in FC between the groups were used as classification features to evaluate whether FC values could reliably differentiate patients with OSA from HC. We applied three supervised machine learning models (SVM, RF, and LR) implemented in Python's scikit‐learn library. The models were trained on a labeled dataset, where known class labels guided the learning process to enable accurate pattern recognition. To enhance model robustness and mitigate overfitting, a 10‐fold stratified cross‐validation strategy was employed, randomly partitioning the data into 10 subsets such that each fold alternated between training and testing roles across multiple iterations (Nunes et al. 2024). Hyperparameters for each model were optimized during this process to improve generalizability. Evaluation metrics comprised accuracy, sensitivity (recall), specificity, precision, F1‐score, and the area under the receiver operating characteristic (ROC) curve (AUC), which together provide a comprehensive and balanced evaluation of model performance. These metrics capture different dimensions of classification effectiveness, from overall correctness (accuracy) to the ability to correctly identify positive cases (sensitivity) and negative cases (specificity). Precision and F1‐score further assess the trade‐off between false positives and false negatives, while the AUC–ROC metric evaluates the model's discrimination capability across various decision thresholds, making it especially informative for imbalanced datasets.

### 2.8 Statistical Analysis

Statistical analyses were performed using SPSS version 23.0 (IBM, USA), with a significance threshold set at *p* < 0.05. Demographic and clinical variables were summarized as mean ± standard deviation. Data normality was assessed using the Kolmogorov–Smirnov test. Group differences in FC were examined using two‐sample *t*‐tests, controlling for head motion as a covariate. Gaussian random field (GRF) theory correction was applied for multiple comparisons (voxel‐level *p* < 0.001, cluster‐level *p* < 0.05, two‐tailed). Partial correlation analyses were conducted to evaluate the associations between mean *z*‐scores of significant FC clusters and clinical variables, with age, body mass index (BMI), years of education, and head motion as covariates. Bonferroni correction was applied to adjust for multiple comparisons in the correlation analyses.

## 3 Results

### 3.1 Demographic and Clinical Characteristics

Statistical comparisons between patients with OSA and HC were conducted using covariates, including head movement parameters, BMI, years of education, and age. Patients with OSA displayed significantly higher values than HC in BMI, AHI, the percentage of total sleep time with SaO_2_ below 90% (SaO_2 _< 90%), N1, arousal index (AI), oxygen desaturation index (ODI), ESS, PSQI, HAMA, and HAMD were significantly higher in patients with OSA (all *p* < 0.05); whereas nadir SaO_2_, mean SaO_2_, sleep efficiency, N3, rapid eye movement (REM), MoCA scores were significantly lower in patients with OSA (all *p* < 0.05). No significant differences were found between the groups in terms of age, total sleep time, N2, years of education, or head movement (all *p* > 0.05). Detailed demographic and clinical data of the two groups are presented in Table 2.

**TABLE 2:** Demographic and clinical data between patients with OSA and HC (mean ± SD).

Characteristic	OSA (*N* = 65)	HC (*N* = 65)	*t*‐value	*p‐*value
Age, years	37.62 ± 10.04	37.46 ± 10.42	0.086	0.932
BMI, kg/m^2^	26.65 ± 3.63	20.93 ± 1.68	11.525	< 0.001^*^
AHI, h^−1^	48.56 ± 18.38	2.27 ± 1.19	20.261	< 0.001^*^
Nadir SaO_2_, %	70.29 ± 11.79	93.58 ± 3.66	−15.217	< 0.001^*^
Mean SaO_2_, %	92.66 ± 4.14	96.86 ± 2.17	−7.242	< 0.001^*^
SaO_2_ <90%	19.99 ± 17.40	0.72 ± 1.41	8.896	< 0.001^*^
Total sleep time, min	385.70 ± 105.09	406.71 ± 23.84	−1.572	0.12
Sleep efficiency, %	0.84 ± 0.19	0.92 ± 0.03	−3.539	0.001^*^
N1,%	27.57 ± 16.02	10.24 ± 3.38	8.53	< 0.001^*^
N2,%	38.22 ± 12.79	41.15 ± 6.15	−1.662	0.1
N3,%	20.56 ± 16.32	30.10 ± 5.51	−4.466	< 0.001^*^
REM,%	13.65 ± 10.79	18.51 ± 5.34	−3.255	0.002^*^
AI, h^−1^	28.24 ± 17.74	11.69 ± 3.03	7.414	< 0.001^*^
ODI	42.95 ± 23.19	1.79 ± 1.07	14.296	< 0.001^*^
Education, years	11.83 ± 3.77	11.32 ± 3.06	0.843	0.401
ESS, scores	10.37 ± 4.33	1.42 ± 1.39	15.866	< 0.001^*^
MoCA scale, scores	24.32 ± 2.90	27.97 ± 1.45	−9.071	< 0.001^*^
PSQI, scores	7.86 ± 3.29	3.66 ± 1.59	9.257	< 0.001^*^
HAMA, scores	9.35 ± 4.87	5.69 ± 3.03	5.148	< 0.001^*^
HAMD, scores	8.72 ± 6.09	3.94 ± 2.88	5.73	< 0.001^*^
Head movement, mm	0.13 ± 0.06	0.12 ± 0.06	1.479	0.142

*Note*: Values are presented as mean ± standard deviation unless otherwise indicated.

Abbreviations: AHI, apnea‐hypopnea index; AI, arousal index; BMI, body mass index; ESS, Epworth sleepiness scale; HAMA, Hamilton Anxiety Scale; HAMD, Hamilton Depression Scale; HC, healthy controls; MoCA, Montreal Cognitive Assessment; *N*, number; ODI, oxygen desaturation index; OSA, obstructive sleep apnea; PSQI, Pittsburgh Sleep Quality Index; REM, rapid eye movement; SaO_2_ < 90%, percentage of total sleep time spent at oxygen saturation less than 90%; SaO_2_, oxygen saturation.

* *p* < 0.05, which was considered statistically significant.

### 3.2 Intergroup Differences in FC of the Subregions of M1

**FIGURE 3:**
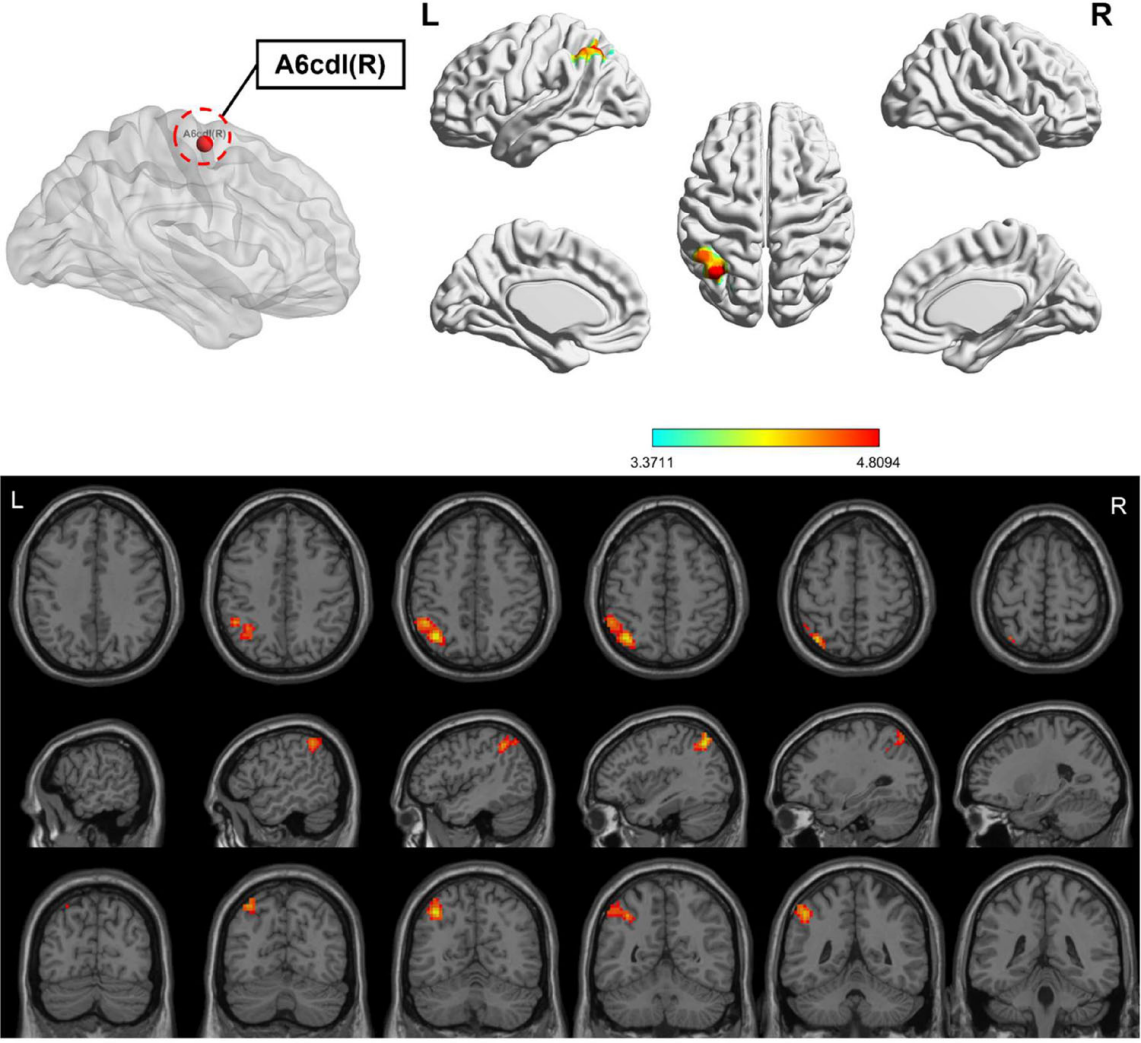
Altered FC between right A6cdl (ROI4) of primary motor cortex subregions and the whole brain in patients with OSA and HC (two‐tailed GRF correction, voxel‐level [*p* < 0.001], and cluster‐level [*p* < 0.05]). ROI, region of interest.

**FIGURE 4:**
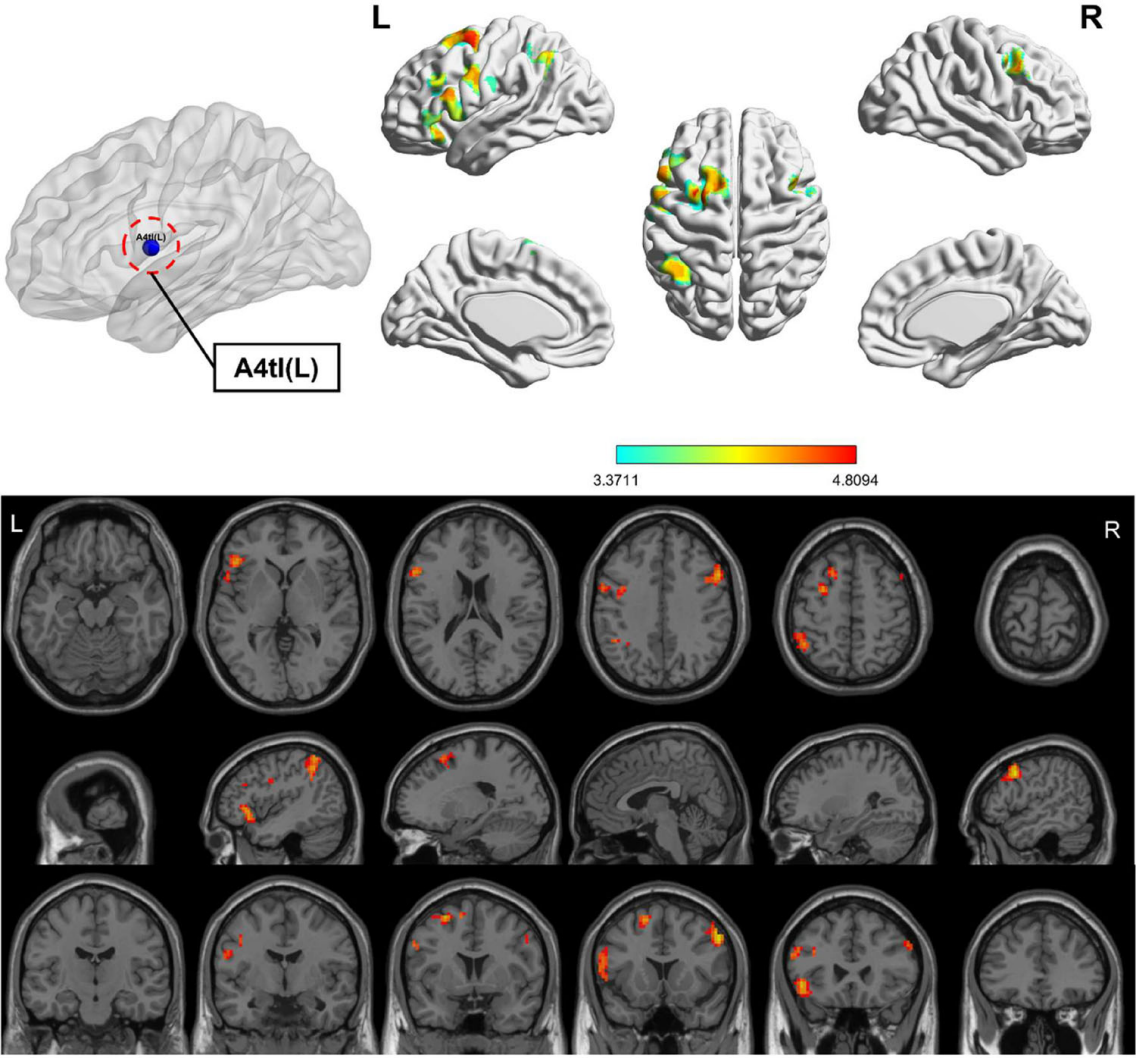
Altered FC between left A4tl (ROI9) of primary motor cortex subregions and the whole brain in patients with OSA and HC (two‐tailed GRF correction, voxel‐level [*p* < 0.001], and cluster‐level [*p* < 0.05]). ROI, region of interest.

**FIGURE 5:**
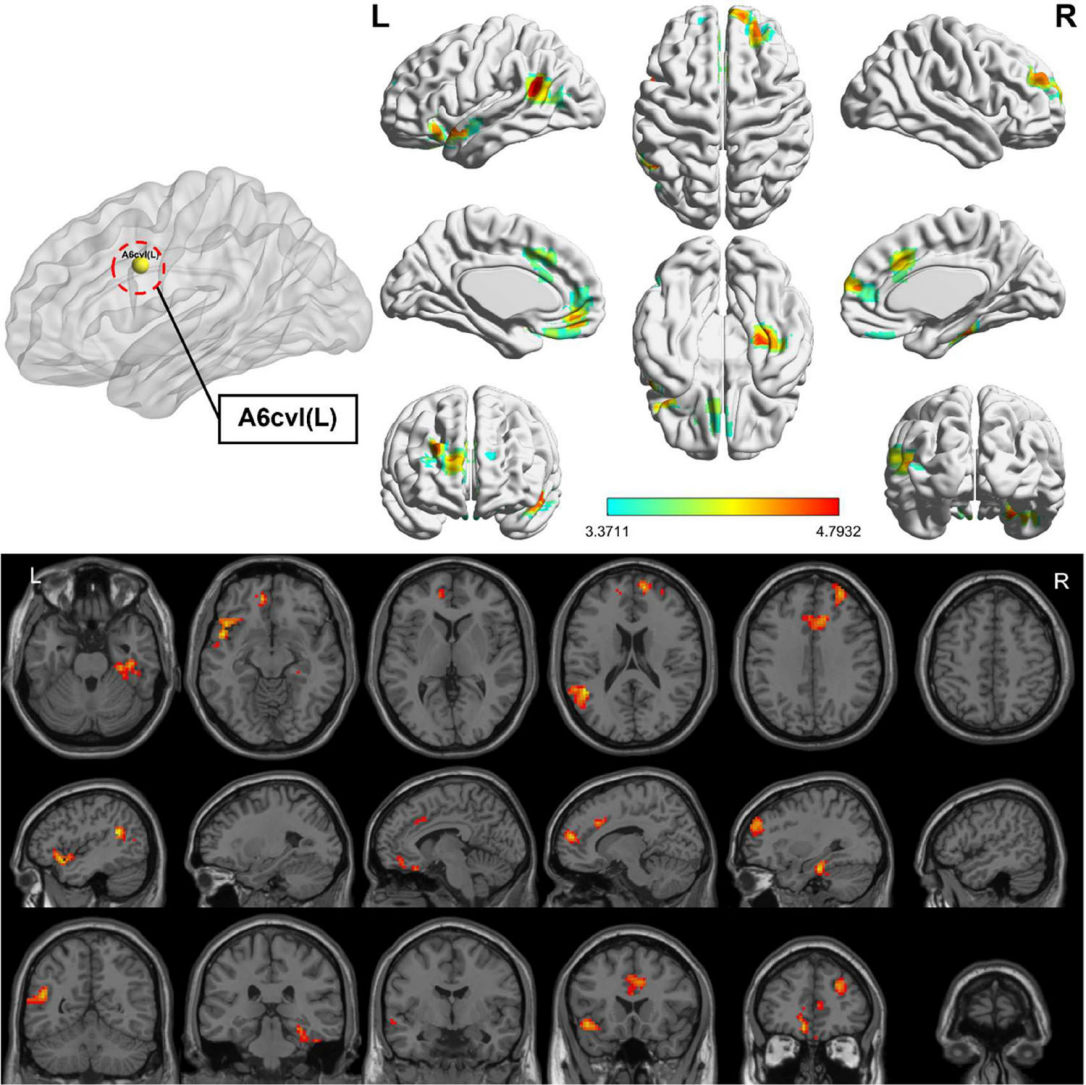
Altered FC between left A6cvl (ROI11) of primary motor cortex subregions and the whole brain in patients with OSA and HC(two‐tailed GRF correction, voxel‐level [*p* < 0.001], and cluster‐level [*p* < 0.05]). ROI, region of interest.

**FIGURE 6:**
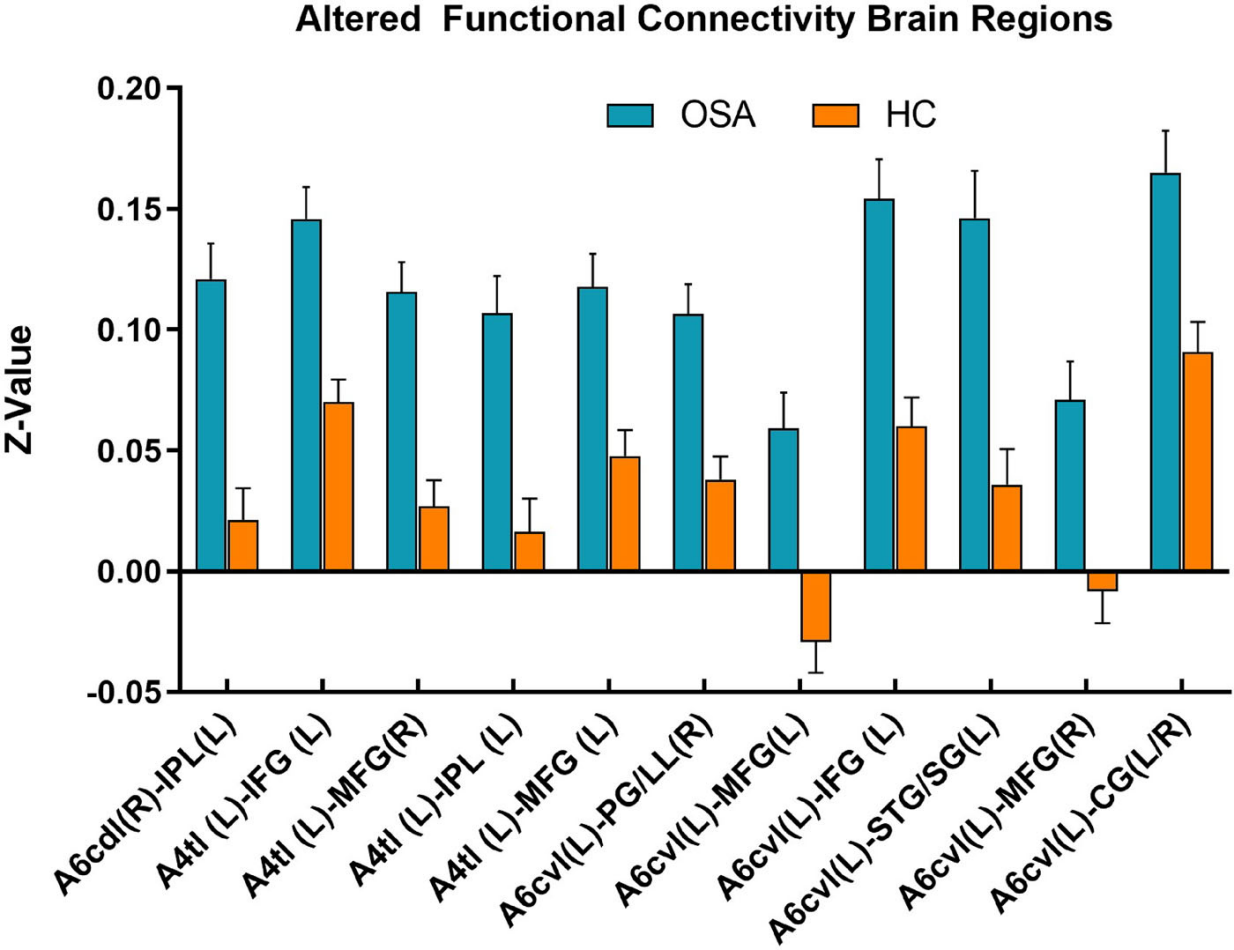
The histogram indicates the mean value of FC between the two groups. Detailed information about these altered regions is provided in Table 3. Values are presented as the mean ± standard error. CG, cingulate gyrus; FC, functional connectivity; IFG, inferior frontal gyrus; IPL, inferior parietal lobule; L, left; MFG, middle frontal gyrus; PG, parahippocampa gyrus; R, right; STG, superior temporal gyrus.

Compared to HC, the OSA group showed enhanced FC between subregions of M1 in various brain areas (Figures 3, 4, 5, 6; Table 3). Specifically, in the OSA group, FC was increased between the right A6cdl and the left inferior parietal lobule (IPL). In the left A4tl, FC was significantly stronger with the left inferior frontal gyrus (IFG), bilateral middle frontal gyrus (MFG), and left IPL. Additionally, the left A6cvl in the OSA group showed increased connectivity with the right parahippocampal gyrus (PG), bilateral MFG, left IFG, left superior temporal gyrus (STG), and right cingulate gyrus (CG).

**TABLE 3:** Brain areas showing functional connectivity differences with M1 subregions between patients with OSA and HC.

Seed‐ ROIs	Brain areas	L/R	Number of voxels	PEAK MNI coordinates			*t*‐value	BA	AAL
				*X*	*Y*	*Z*			
A6cdl (R)	IPL	L	227	−36	63	51	4.955	7L	Parietal_Inf
A4tl (L)	IFG	L	271	−48	24	−3	4.429	47L	Frontal_Inf_Orb
	MFG	R	115	54	15	39	4.809	9R	Frontal_Mid
	IPL	L	128	−45	−51	57	4.328	40L	Parietal_Inf
	MFG	L	114	−27	3	57	4.706	6L	Frontal_Mid
A6cvl (L)	PG	R	110	30	−24	−24	4.638	20R	ParaHippocampal
	MFG	L	103	−9	45	−12	4.373	11L	Frontal_Med_Orb
	IFG	L	118	−48	9	−9	4.793	48L	Temporal_Pole_Sup
	STG	L	164	−45	−51	21	4.789	21L	Temporal_Mid
	MFG	R	211	12	57	15	4.479	10R	Frontal_Sup_Medial
	CG	L/R	121	9	24	33	4.255	32R	Cingulum_Mid

*Note*: All clusters were reported with a voxel‐level threshold of *p *< 0.001, and cluster‐level of *p *< 0.05, two‐tailed.

Abbreviations: BA, Brodmann area; CG, cingulate gyrus; HC, healthy controls; IFG, inferior frontal gyrus; IPL, inferior parietal lobule; L, left; MFG, middle frontal gyrus; MNI, Montreal Neurological Institute; PG, parahippocampa gyrus; R, right; ROI: region of interest; STG, superior temporal gyrus.

### 3.3 Correlational Analysis between FC Showing Group Differences and Clinical Variables in Patients with OSA

**FIGURE 7:**
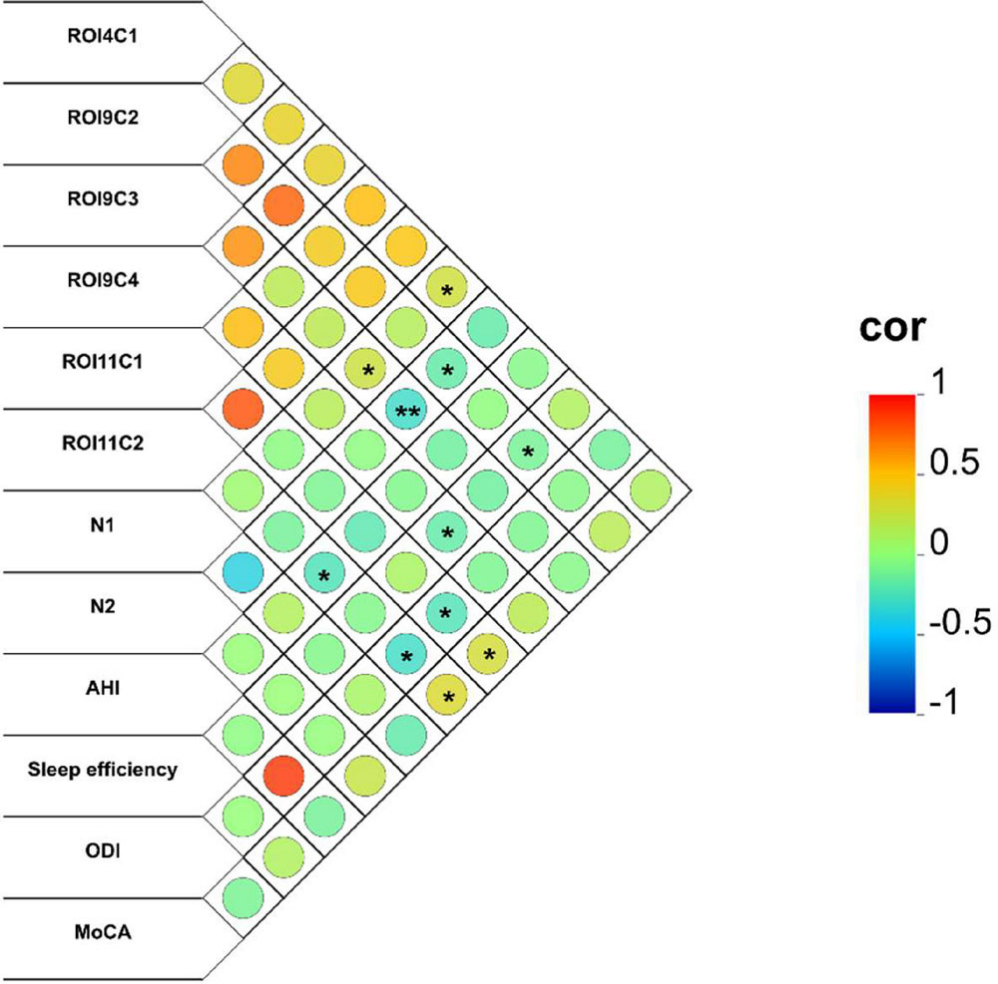
The correlations between the altered FC values and clinical variables in abnormal brain regions among patients with OSA. In patients with OSA, there was a significant correlation between FC values with intergroup differences (patients with OSA vs. HCs) and clinical assessments. * and ** denote existing statistical difference before and after Bonferroni correction. AHI, apnea‐hypopnea index; C1‐4, cluster 1‐4; FC, functional connectivity; L, left; MoCA, Montreal Cognitive Assessment; N1/2, sleep stage 1/2; ODI, oxygen desaturation index; OSA, obstructive sleep apnea; R, right; ROI, region of interest.

Correlational analysis, controlled for years of education, BMI, head movement, and age, revealed significant relationships between altered FC patterns and clinical metrics in patients with OSA, which was shown in Figure 7 and Table 4. Specifically, FC between the right A6cdl and left IPL showed a positive correlation with N1 sleep. In contrast, FC between the left A4tl and right MFG was negatively correlated with N2 and sleep efficiency. The FC between the left A4tl and left IPL was positively correlated with N1, whereas a robust negative correlation with N2 remained significant after the Bonferroni correction. Additionally, the FC between the left A4tl and left MFG was negatively associated with sleep efficiency. The left A6cvl‐right PG connectivity was positively correlated with MoCA scores and negatively correlated with ODI. Additionally, the FC between the left A6cvl and left MFG was negatively correlated with both AHI and ODI, alongside a negative correlation with MoCA scores.

**TABLE 4:** Significant associations between cerebellar FC and clinical characteristics in patients with OSA.

ROI	FC between brain regions	Clinical characteristic	*p*‐value	*r*‐value
ROI4C1	A6cdl (R)–IPL (L)	N1	0.036	0.27
ROI9C2	A4tl (L)–MFG (R)	N2	0.013	−0.315
	A4tl (L)–MFG (R)	Sleep efficiency	0.033	−0.274
ROI9C3	A4tl (L)–IPL (L)	N1	0.035	0.273
	A4tl (L)–IPL (L)	N2	0.003	−0.378^a^
ROI9C4	A4tl (L)–MFG (L)	Sleep efficiency	0.013	−0.318
ROI11C1	A6cvl (L)–PG (R)	MoCA	0.044	0.258
	A6cvl (L)–PG (R)	ODI	0.032	−0.275
ROI11C2	A6cvl (L)–MFG (L)	AHI	0.044	−0.259
	A6cvl (L)–MFG (L)	ODI	0.012	−0.32
	A6cvl (L)–MFG (L)	MoCA	0.006	−0.35

^a^Existing statistical difference after Bonferroni correction.

Abbreviations: AHI, apnea‐hypopnea index; C1‐4, cluster 1‐4; FC, functional connectivity; IPL, inferior parietal lobule;L, left; MFG, middle frontal gyrus; MoCA, Montreal Cognitive Assessment; N1/2, sleep stage 1/2; ODI, oxygen desaturation index; OSA, obstructive sleep apnea; PG, parahippocampa gyrus; R, right; ROI: region of interest.

### 3.4 SVM Classification Results

**FIGURE 8:**
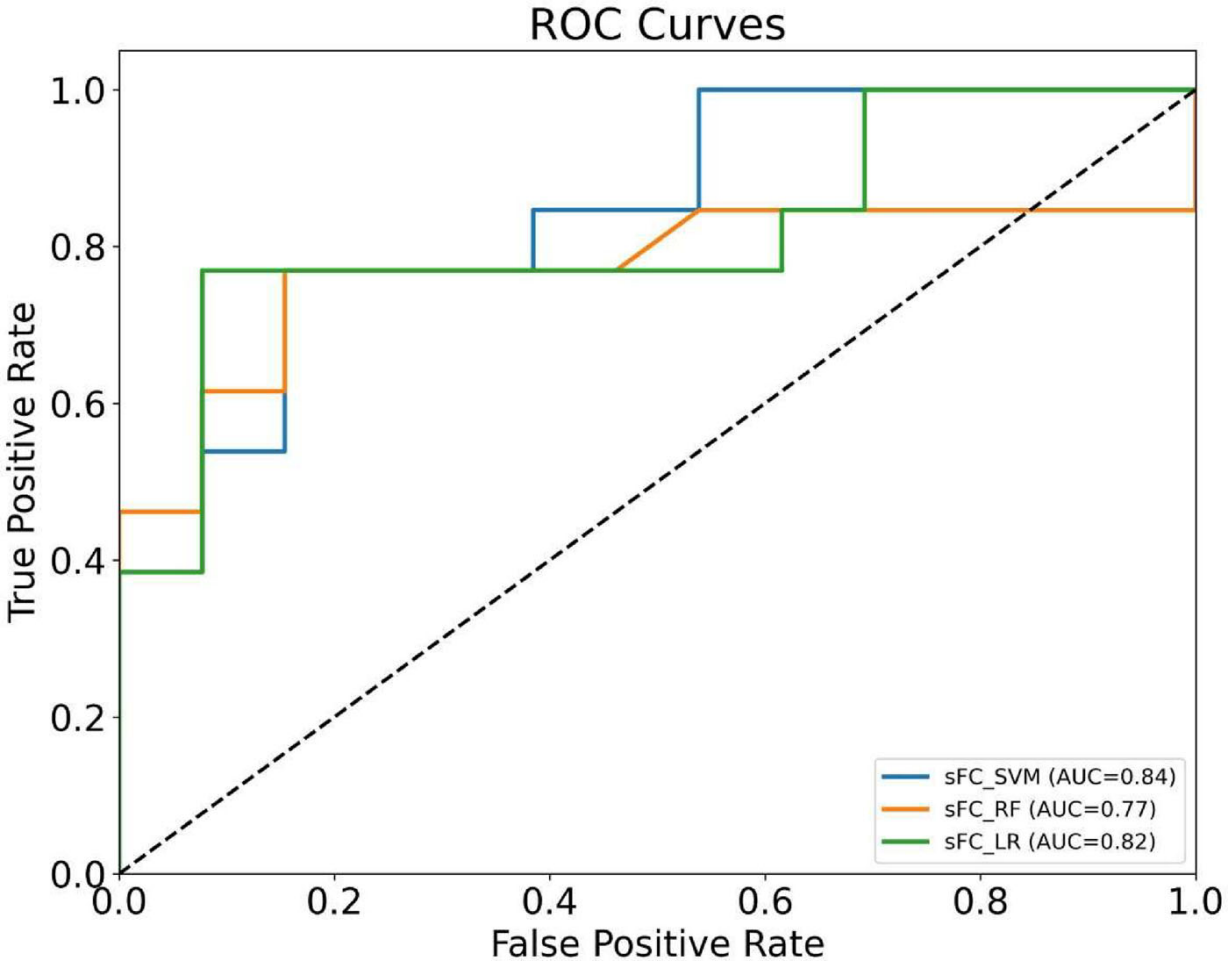
ROC curves for classifying OSA and HC using static FC features with SVM, RF, and LR models. SVM showed the highest AUC (0.84), followed by LR (0.82) and RF (0.77). AUC, area under the ROC curve; LR, logistic regression; RF, random forest; ROC, receiver operating characteristic; sFC, static functional connectivity; SVM, support vector machine.

Significant differences in FC between subregions of M1 and other brain regions were observed between patients with OSA and HC. As shown in Figure 8 and Table 5, classification based on FC features yielded varying results across different machine learning models. Among the three approaches, SVM exhibited the highest overall performance, with an AUC of 0.84, accuracy of 0.77, precision of 0.82, specificity of 0.85, and F1‐score of 0.75. These results suggest that SVM provided the most favorable balance across key metrics. LR achieved a slightly lower AUC (0.82), with notably higher sensitivity (0.77), but lower specificity (0.69), indicating that it was more likely to detect positive cases at the expense of increased false positives. RF showed comparable accuracy (0.73) and balanced sensitivity and specificity (both 0.69 and 0.77, respectively), but its AUC (0.77) was lower than both SVM and LR.

**TABLE 5:** Performance evaluation of classifier using different model.

Feature	Model	SVM	LR	RF
FC	AUC	0.84	0.82	0.77
	Accuracy	0.77	0.73	0.73
	Precision	0.82	0.71	0.75
	Sensitivity	0.69	0.77	0.69
	Specificity	0.85	0.69	0.77
	F1‐score	0.75	0.74	0.72

Abbreviations: AUC, area under the curve; FC, functional connectivity; LR, logistic regression; RF, random forest; SVM, support vector machine.

## 4 Discussion

This study, utilizing the Human Brainnetome Atlas, is the first to systematically examine FC changes between subregions of M1 and whole‐brain regions in patients with OSA, shedding light on the neuropathological mechanisms of OSA and potentially offering novel insights into targeted therapeutic strategies. Our findings indicate that the FC between several subregions of M1 and whole‐brain regions is increased to varying extents in patients with OSA. These neurological alterations in OSA suggest that these FC changes may serve an adaptive, compensatory role. Patients with OSA may experience impaired cognitive and motor functions, along with altered resting‐state functional activity in specific brain regions.

### 4.1 Enhanced FC in the Right A6cdl and Left A4tl: Implications for Motor Control, Spatial Perception, and Executive Functions

Studies have documented cognitive impairments in patients with OSA, including memory deficits, inattention, and delayed responses (Li, Liu, et al. 2024; Sui et al. 2024).In this study, enhanced FC between the right A6cdl and the left IPL in patients with OSA suggests potential disruptions in motor control and spatial perception. The IPL plays a crucial role in integrating spatial information and coordinating movement, and the enhanced FC between right A6cdl and IPL may reflect a compensatory mechanism within patients with OSA as they adapt to deficits in motor control and spatial perception resulting from impaired neural function (Heyes and Catmur 2022; Huang et al. 2023). This strengthened connectivity likely represents the brain's attempt to preserve motor and cognitive functions by reorganizing FC in response to OSA‐related neurological disruptions (Hok et al. 2024).

Furthermore, FC changes in the left A4tl exhibited a more intricate pattern. In addition to enhanced connectivity with the left IPL, the left A4tl also showed significantly increased FC with the MFG and IFG. This pattern suggests extensive impairments in executive function and spatial cognition in patients with OSA. The MFG is crucial for working memory and attentional control, allowing individuals to sustain focus and manage multiple streams of information when performing complex tasks (Bramen et al. 2023). Meanwhile, the IFG contributes to inhibitory control, decision‐making, and language processing, supporting robust decision‐making by enabling individuals to suppress impulsive responses and make rapid judgments. Thus, the aberrant FC between A4tl and these regions may indicate significant difficulties for patients with OSA in task‐switching, sustained attention, and complex decision‐making. These functional changes not only affect executive function but also increase potential safety risks for patients with OSA in high‐load task environments.

Indeed, studies have indicated a significantly elevated risk of car accidents in patients with OSA compared to the general population (Constante et al. 2023; Simpamba et al. 2023). These FC alterations directly impact the driving safety of these patients, for whom driving represents a complex perceptual‐motor task that requires rapid spatial information processing and precise responses (Stojan and Voelcker‐Rehage 2021). Enhanced right A6cdl‐IPL connectivity may reduce dynamic spatial processing efficiency in patients with OSA, increasing the risks of delayed responses and compromised spatial judgment. Additionally, abnormal FC between the left A4tl and both the MFG and IFG affects executive function demands critical for driving, such as sustained attention, suppression of unnecessary reactions, and rapid decision‐making. With the central role of the IFG in reaction inhibition and decision‐making, impaired functionality in these areas may lead patients with OSA to make suboptimal decisions in emergencies, increasing driving‐related risks.

Patients with OSA may perform poorly in driving situations that require quick and precise responses, such as sudden braking or obstacle avoidance. Furthermore, these patients frequently report symptoms of distractibility and mental fatigue (Chervin 2000; Marrone et al. 2013), which align with observed FC alterations in the MFG and CG. Individuals with OSA may expend more neural resources to sustain attention while driving, leading to heightened fatigue over extended driving periods and reduced responsiveness. Mental fatigue further diminishes cognitive and emotional regulation, causing declines in alertness, attention span, and short‐term memory (Dong et al. 2024). This cumulative neural burden not only impacts everyday activities for patients with OSA but also escalates risks in tasks requiring sustained concentration, such as driving. Extensive FC reorganization in OSA may compromise brain efficiency in information processing, further affecting daily functioning and driving safety. Therefore, understanding FC alterations and their impact on driving risks in patients with OSA is crucial for developing targeted intervention and rehabilitation strategies. Notably, treatments for OSA, such as continuous positive airway pressure (CPAP) therapy, hold significant potential to restore normative neural FC patterns and mitigate traffic accident risks.

### 4.2 Enhanced FC in the Left A6cvl and Its Implications on Emotion Regulation, Auditory Processing, and Memory Integration

The increased FC in the left A6cvl subregion extends to multiple critical regions involved in emotional, auditory, and memory processing, including the right PG, bilateral MFG, left IFG, STG, and bilateral CG. Enhanced A6cvl‐PG connectivity may contribute to the memory issues frequently reported by patients with OSA, as the PG plays an essential role in memory integration. Disruptions in this connection may underlie difficulties with long‐term memory and quick retrieval of information (Patel and Chong 2021). This impairment often surfaces in everyday tasks as decreased productivity or learning challenges, particularly in contexts requiring complex information processing, where patients might expend additional neural resources to sustain memory function (Naëgelé et al. 2006). Medial frontal lobe activation is often linked to self‐referential processing and social cognition (Merle et al. 2021), which suggests that OSA may influence social competencies and self‐perception. Additionally, increased connectivity between A6cvl and the STG may negatively impact auditory processing and language comprehension. The STG is central to language decoding and auditory information processing; thus, these changes may hinder the ability of patients with OSA to concentrate and quickly interpret language in complex auditory settings (e.g., noisy environments or multi‐speaker conversations). This impairment is not limited to language comprehension but may also affect performance in complex conversations or linguistic tasks, aggravating social difficulties, particularly in scenarios requiring attention to multiple conversations. Enhanced FC in the CG, especially in the anterior and mid‐cingulate regions, likely correlates with mood disorders (e.g., anxiety and depression) that are commonly observed in patients with OSA. These areas are closely associated with emotion regulation and cognitive control, and patients with OSA may face challenges in emotional coping and stress management owing to such connectivity abnormalities. Increased CG activity indicates that the brains of patients with OSA may require additional neural resources to manage emotional stress, further contributing to emotional instability and mental fatigue. This compensatory activity may also reflect the need for greater efforts to maintain emotional and cognitive balance in daily life, making mental and physical fatigue more pronounced after prolonged work or study sessions.

#### 4.2.1 FC Changes and Associations with Clinical Characteristics

Our findings revealed that, following Bonferroni correction, there was a significant negative correlation (*p* < 0.05) between the FC of the left A4tl and left IPL and N2. This correlation provides insights into the neuropathological mechanisms in patients with OSA. N2 constitutes a substantial portion of non‐REM sleep and is essential for memory consolidation and neuroplasticity, particularly through the generation of sleep spindles and K‐complexes (Wright et al. 2023). However, N2 is often significantly disrupted in patients with OSA, indicating a disturbed sleep architecture (Zhu et al. 2023). The A4tl subregion in the primary motor cortex primarily regulates motor control of the tongue and laryngeal muscles, while the IPL contributes to higher order cognitive functions such as spatial perception and attentional allocation (Cai et al. 2024). The observed negative correlation between A4tl and IPL FC may reflect dysregulation in upper airway muscle control during N2 in patients with OSA. With airway obstruction, patients with OSA may struggle to regulate the activity of tongue and throat muscles during sleep, leading to breathing difficulties that compromise N2 quality (A. Chen et al. 2023). This weakened FC may result from an imbalance in upper airway muscle control, which may further impair memory consolidation and cognitive functions in patients. This finding indicates that OSA is more than a sleep breathing disorder; it also disrupts normal sleep architecture by altering FC in the M1 region, particularly in its connection to the IPL. Given the critical role of N2 in memory and cognitive function, FC abnormalities in the M1 region may contribute to cognitive decline in patients with OSA.

Additionally, although some correlations from uncorrected analyses have lower statistical significance, they nonetheless offer valuable insights into FC changes in patients with OSA. For instance, the positive correlation between A6cdl‐IPL FC and N1 sleep, as well as the negative correlation between A4tl‐MFG FC and sleep efficiency, further supports the potential role of subregions of M1 in sleep regulation in patients with OSA. These findings suggest that OSA affects not only the sleep architecture of patients but may also influence the overall sleep efficiency and sleep initiation processes. These results highlight the intricate relationships between FC changes, clinical features of OSA, and cognitive performance. Although the uncorrected results require cautious interpretation, they underscore important future research directions, advocating for deeper exploration of the multidimensional impacts of sleep, cognition, and respiratory function in patients with OSA. Future studies with larger sample sizes and more rigorous statistical methods are necessary to validate these findings, elucidate the complex pathophysiology of OSA, and substantiate clinical interventions.

### 4.3 Prospects of Machine Learning in Patients With OSA

This study employed the SVM algorithm to successfully differentiate patients with OSA from HC based on the FC features of the subregions of M1. SVM is a promising analytical method that is widely used across various diseases and shows robust classification performance (Bu et al. 2024; Naqvi et al. 2023). Our findings indicated strong accuracy, sensitivity, and specificity, suggesting that FC alterations in subregions of M1 may serve as an effective biomarker for diagnosing OSA. Clinically, this finding is important as it provides an objective supplementary tool to traditional PSG, highlighting the diagnostic value of FC indices in subregions of M1 for OSA. In addition to SVM, we also applied LR and RF models, which yielded comparable yet slightly lower performance, further supporting the feasibility of multiple machine learning approaches for FC‐based classification. These results suggest that multi‐model approaches may enhance diagnostic reliability in future applications.

Machine learning classification studies based on MRI data have already been broadly applied in OSA research (Pang et al. 2023; Xie et al. 2022). Our findings further advance this field by highlighting the diagnostic value of M1‐specific FC patterns, which have not been extensively explored in previous studies. In particular, the superior performance of the SVM model underscores the potential of M1 FC features as reliable neuroimaging biomarkers for OSA. However, certain challenges remain in implementing this approach in clinical practice. First, large‐scale, multicenter validation is needed to confirm the model's robustness across diverse populations. Second, standardization of FC acquisition and preprocessing pipelines is essential to facilitate reproducibility and clinical adoption. Third, differences in MRI protocols and machine learning pipelines across institutions may hinder generalizability, necessitating the development of harmonized processing frameworks. Finally, assessing the cost‐effectiveness of this approach is essential to determine its feasibility in clinical settings. Despite these challenges, our findings open up promising research avenues in OSA diagnosis. Future studies should explore the combination of FC features with other clinical and imaging indicators to enhance diagnostic accuracy and comprehensiveness. Additionally, this approach may prove useful for monitoring treatment effects and evaluating the impact of various treatment regimens on brain function in patients with OSA.

### 4.4 Limitations

This study had some limitations. First, the absence of motor assessments restricted our interpretation of changes in motor function. Future studies should incorporate motor‐related evaluation tools, such as motor coordination and reaction speed scales, to better evaluate the impact of OSA on motor capabilities. Second, due to the prevalence characteristics of OSA, the limited number of female participants restricted sex‐based analysis. Future studies should include a larger number of female and pediatric patients to comprehensively examine OSA across various populations. Our analysis focused primarily on predefined ROI, which may have led to the exclusion of other potentially relevant brain regions. Future studies should consider whole‐brain analysis methods and include appropriate control conditions to further clarify the functional significance of these activation patterns. In addition, we did not apply a formal feature selection algorithm, which may have limited the optimization of classifier performance; future studies with larger samples should allow the integration of advanced selection methods to refine FC‐based predictive modeling. Lastly, the modest sample size may have limited statistical power to detect subtle effects; future studies with larger cohorts are needed.

## 5 Conclusion

This study revealed significant effects of OSA on brain functional networks, particularly in motor control through FC changes in the subregions of M1. These alterations correlate with upper airway muscle dysfunction and cognitive impairments, notably increasing the risk of motor vehicle accidents. Furthermore, the links between the subregions of M1, sleep architecture, and cognitive function highlight that OSA is not merely a sleep‐related breathing disorder but a complex condition with multisystem involvement. Our results provide new perspectives for OSA management, emphasizing the need for comprehensive treatment approaches that address both motor and cognitive functions to improve patient safety, especially during driving. The high accuracy of SVM classification based on FC patterns demonstrates the potential utility of these connectivity changes as neuroimaging biomarkers for OSA diagnosis. In summary, by systematically investigating FC alterations in subregions of M1 in patients with OSA, this study aims to uncover specific effects of OSA on the motor control system, provide new perspectives for OSA diagnosis, prognosis, and treatment, and potentially contribute to improving the quality of life for individuals affected by OSA.

## Author Contributions

**Lifeng Li**: writing – review and editing, validation, software, methodology, data curation, writing – original draft. **Qimeng Shi**: data curation. **Bowen Fang**: investigation, supervision. **Yuting Liu**: methodology, validation. **Xiang Liu**: supervision, investigation, visualization. **Yongqiang Shu**: investigation, validation. **Yingke Deng**: visualization, project administration, supervision. **Yumeng Liu**: formal analysis, investigation. **Haijun Li**: data curation, writing – review and editing, writing – original draft, funding acquisition. **Junjie Zhou**: investigation, software, resources. **Dechang Peng**: conceptualization, investigation, funding acquisition.

## Ethics Statement

Written consent was obtained from all the participants with potentially identifiable images or data. The study was approved by the Ethics Committee of the First Affiliated Hospital of Nanchang University.

## Peer Review

The peer review history for this article is available at https://publons.com/publon/10.1002/brb3.70698

## Data Availability

The original contributions presented in the study are included in the article. Further inquiries can be directed to the corresponding author.
